# Does the Dark Triad of Personality Predict Corrupt Intention? The Mediating Role of Belief in Good Luck

**DOI:** 10.3389/fpsyg.2016.00608

**Published:** 2016-04-28

**Authors:** Huanhuan Zhao, Heyun Zhang, Yan Xu

**Affiliations:** School of Psychology, Beijing Normal UniversityBeijing, China

**Keywords:** Machiavellianism, narcissism, psychopathy, belief in good luck, corruption

## Abstract

The current study is the first attempt to examine the association between the Dark Triad of personality (i.e., Machiavellianism, narcissism, and psychopathy) and corruption through a mediator—belief in good luck. Based on Ajzen's theory of planned behavior, we assumed that individuals with Dark Triad would be more likely to engage in corruption as a result of belief in good luck. In Study 1, a set of hypothetical scenarios was used to assess the bribe-offering intention and the corresponding belief in good luck. Results indicated that while the Dark Triad of personality positively predicted bribe-offering intention, it was mediated by the belief in good luck in gain-seeking. In Study 2, we presented participants with some hypothetical scenarios of bribe-taking and the corresponding belief in good luck. Findings revealed that the Dark Triad of personality was positively related to bribe-taking intention; the relationship between narcissism and bribe-taking intention, and that between psychopathy and bribe-taking intention was mediated by the belief in good luck in penalty-avoidance. However, this belief in good luck did not mediate the relationship between Machiavellianism and bribe-taking intention. These results hold while controlling for demographic variables, dispositional optimism, and self-efficacy. Taken together, this study extended previous research by providing evidence that belief in good luck may be one of the reasons explaining why people with Dark Triad are more likely to engage in corruption regardless of the potential outcomes. Theoretical and practical implications were discussed.

## Introduction

Corruption exists widely. It is commonly defined as deviant behavior that deliberately breaks legal or moral norms and abuses public authority or resources for personal gain (He, 2000; Lindgreen, 2004; Rabl and Kuhlmann, 2008). It impairs political stability, damages economic growth, misallocates public resources, hinders normal upward social mobility, increases social inequality, undermines people's trust in government, and lowers moral standards in a society (He, 2000; Lu and Gunnison, 2003; Alesina and Angeletos, 2005; Sobhani and Bechara, 2011). Since corruption harms a society tremendously, a thorough understanding of it followed by the proper counter measures becomes extremely important.

When it comes to corruption, the following important questions are often raised first: What kinds of people are more likely to act corruptly? What type of personality they possess that leads them to gain profits form corruption at the expense of others? Why do these people tend to engage in corruption more often than others? Though previous research tried to uncover the occurrence of corruption at both macro (Treisman, 2000; Blackburn and Forgues-Puccio, 2009) and micro level (Jaber-López et al., 2014), and found that corruption was a result of interactions among various variables (e.g., political, social, economic, or psychological factors), yet to date, little research has explored the wider range of personality traits potentially associated with corrupt behaviors. A growing body of evidence suggested that the Dark Triad of personality (i.e., Machiavellianism, narcissism, and psychopathy) was associated with unethical behaviors (Egan et al., 2015; Azizli et al., 2016; Roeser et al., 2016). We reckoned that this association may extend to corrupt behaviors. Thus, the first purpose of this study is to examine whether people with Dark Triad are more likely to engage in corruption.

Furthermore, despite the increasing evidence justifying the effects of the Dark Triad traits on various deviant behaviors, scant attention has been given to the underlying mechanism and processes through which this relationship occurs. Therefore, the second purpose of this study is to explore the psychological mechanism that underlies the association between the Dark Triad traits and corruption. According to Ajzen's theory of planned behavior, behavioral dispositions, such as personality traits and social attitudes, played a critical role in predicting cognitive beliefs (e.g., behavioral beliefs, normative beliefs, and control beliefs), which in turn explain human behaviors (Fishbein and Ajzen, 1975; Ajzen, 1991). Ajzen (1991) proposed that personality traits influenced behavior indirectly through cognitive beliefs. Belief in good luck, as an irrational cognitive belief, thus may be affected by one's particular personality trait. Additionally, belief in good luck has been proved to shape one's behaviors (Chiu and Storm, 2010). Accordingly, we assume that the relationship between the Dark Triad of personality and corruption is mediated by belief in good luck.

### The Dark Triad of personality and corruption

The Dark Triad consists of three antisocial personality traits: Machiavellianism, narcissism, and psychopathy (Paulhus and Williams, 2002). Machiavellianism is portrayed as calculated manipulation, duplicity, cunningness, and a disregard for morality (Hodson et al., 2009; Rauthmann and Kolar, 2013; Djeriouat and Tremoliere, 2014). Narcissism is characterized by optimistic egotism (Jones, 2013), and is positively correlated with self-centeredness, sense of superiority, entitlement, vanity, and grandiosity (Crysel et al., 2013; Rauthmann and Kolar, 2013; Buelow and Brunell, 2014). Narcissists often pursue immediate profits at the expense of others' interests (Lakey et al., 2008; Foster et al., 2009). Psychopathy is linked to high impulsivity, callousness, and socially aversive behaviors (Hodson et al., 2009; Rauthmann and Kolar, 2013). According to the life history theory, individuals with Dark Triad manifest the fast life strategy characterized by a disregard for social rules, short-term thinking, and extensive future-discounting related behaviors (Jonason et al., 2010, 2012). These traits are positively related to numerous deviant behaviors like gambling (Jones and Figueredo, 2013), lying and deception (Azizli et al., 2016), cyber-aggression (Pabian et al., 2015), and white-collar crime (Egan et al., 2015). Since corruption is a deviant behavior and can be a criminal offense (Rabl and Kuhlmann, 2008), we posit that the Dark Triad of personality may be positively associated with corrupt behaviors.

Additionally, corruption is based on the exchange between at least two partners, i.e., the bribe giver and taker strike a deal, often by putting their personal interests ahead of others' (Rabl and Kuhlmann, 2008). Research indicated that people with Dark Triad fit this description. Each trait in Dark Triad may have a unique set of features, but all three have something in common as well. First, all three traits are related to the willingness to gain profit at the expense of others (Jones, 2013). Individuals who exhibit high Dark Triad tendencies employ devious means to achieve personal goals with little concern for others' interests (Linton and Power, 2013). Second, the common features of the Dark Triad, such as manipulation, callousness, and selfishness, positively predict deliberate toxic behaviors (O'Boyle et al., 2012; Jones and Figueredo, 2013). Machiavellians often manipulate others for personal gain, against others' welfare (Tang et al., 2008). Narcissists are relentless and toxic when they have power (Schmidt, 2008). A recent study found that individuals with psychopathy share many behavioral characteristics observed in patients suffering from ventromedial prefrontal cortex and amygdala lesions. This finding served as a neuroscientific evidence to explain why psychopaths engage in corrupt and immoral behaviors (Sobhani and Bechara, 2011). Therefore, considering the generally antisocial and socially undesirable nature of the Dark Triad traits, we made an assumption that the Dark Triad traits would predict greater intention to engage in corruption (Hypothesis 1).

### The mediating role of belief in good luck

As pointed out previously, people with Dark Triad may tend to engage in corrupt behaviors. The question is, what motivates these “Dark Triad” people to ignore the law and involve in corruption? According to the theory of planned behavior, belief in good luck can provide a new perspective to look at the reasons behind the Dark Triad people engaging in corruption.

#### Belief in good luck

We can easily observe in daily life the following phenomena: some people cannot stop a gambling game, for they believe that they will have a good luck to win the game, and the good luck makes them believe that their chance of winning will be high (e.g., 70%), despite the actual winning probability is very low (e.g., 5%) and they have lost many times. Or, some people believe that they will have a good luck which makes them not being caught or have low chances of being caught (e.g., 5%) if they cheat just once in an examination, despite the actual probability of being caught is very high (e.g., 60%) and many cheaters have been caught. This irrational belief, often closely related to the negative or deviant behaviors, is named “belief in good luck” in this study. Belief in good luck is an irrational cognition about luck (Day and Maltby, 2003). It can increase ones' unrealistic optimism and self-efficacy (Darke and Freedman, 1997a; Damisch et al., 2010), and affect their future expectations (Darke and Freedman, 1997b).

Belief in good luck is often manifested as blindness in making decisions on probability events, especially with reference to the events involving deviant behaviors (Chiu and Storm, 2010). It should be noted that there are two outcomes of negative or deviant behaviors: positive-valence and negative-valence outcomes. In the above examples, people are attracted to the deviant behaviors of gambling and cheating through different mechanisms. While the former involves overrating the probability of a positive-valence outcome (i.e., winning the game) despite its actual probability is low, the latter involves underrating the probability of a negative-valence outcome (i.e., being caught) despite its actual probability is high. If the actual probability of a positive-valence outcome is very low and the actual probability of a negative-valence outcome is very high, but people still irrationally believe that they will have a good luck, and the corresponding good luck makes them irrationally believe that they are more likely to experience a positive-valence outcome and less likely to experience a negative-valence outcome, then this is the effect of what we call “belief in good luck.” When good luck was thought of as a personal quality possessed by persons, it could provide a perceived ability that can be used to exert control over what otherwise may consider a chance event (Wohl and Enzle, 2002). Accordingly, belief in good luck exerts its influence on negative or deviant behavior via two mechanisms: (1) irrationally overestimating the probability of a positive-valence outcome when its actual probability is very low; and (2) irrationally underestimating the probability of a negative-valence outcome when its actual probability is very high.

Obviously, the operational definition of belief in good luck is somewhat similar to the prospect theory, which also emphasizes decision-making and probability estimation (Tversky and Kahneman, 1981, 1992). The prospect theory suggests that people tend to overrate the small probabilities and underrate the moderate and large probabilities regardless of the nature of the event (e.g., good or deviant behavior) and the valence of the event outcome (i.e., positive or negative valence; Tversky and Kahneman, 1981, 1992; Kusev et al., 2009). Nevertheless, belief in good luck takes into account of the outcome valence of deviant event as well as its actual probability. Additionally, we should point out that it is plausible to make the inference that when the probability of a positive-valence outcome is very high, people may still overrate it, and when the probability of a negative-valence outcome is very low, people may still underrate it, despite the respective degrees of overestimation and underestimation may be very small. It is reasonable and natural for people to expect that they will experience a positive-valence outcome when its probability is very high and will not suffer from a negative-valence outcome when its probability is very low. Evidently, these two cases not only contradict the prospect theory (Tversky and Kahneman, 1981, 1992), but also in line with people's rational expectations, therefore, are not considered as an irrational belief in good luck.

Based on the prospect theory and the operational definition of belief in good luck, we used the adapted research paradigm of “*objective probability event*-*subjective probability estimation*” to measure one's belief in good luck in corruption. Here the objective probability event means the actual probability of deviant event outcomes, whereas the subjective probability estimation means people's irrational overestimation and underestimation. We also employed two different outcome valences of the forms of corruption in this study to verify the related mechanisms of belief in good luck. As to bribe-offering, in order to gain unfair advantages over others, one may offer a bribe to someone who is in power. However, in China, since bribe-offering is legally much less penalized than bribe-taking (Wang and Wu, 2012), we focused on the likelihood of seeking gains via bribe-offering (a positive-valence outcome of deviant behavior). We contended that some people may have the lucky belief and tend to irrationally overestimate the probability of seeking personal advantages via bribe-offering *(namely belief in good luck in gain-seeking)* (Study 1). Additionally, since bribe-taking behaviors in China face much more severe penalties and involvement in bribe-taking is becoming increasingly risky (Gong, 2002; Lu and Gunnison, 2003), the factor to focus on here is the probability of being penalized in bribe-taking (a negative-valence outcome of deviant behavior). We speculated that some people may hold the lucky belief and have a tendency to irrationally underestimate the likelihood of being penalized for bribe-taking *(namely belief in good luck in penalty-avoidance)* (Study 2).

#### The Dark Triad of personality and belief in good luck

The theory of planned behavior suggests that personality traits play an important role in predicting cognitive beliefs (Fishbein and Ajzen, 1975; Ajzen, 1991). People's personality influences how they perceive and evaluate things around them (Andre, 2006; Jibeen, 2015). If a personality trait toward cognitive irrationality is rooted largely in innate or biological differences, it is more likely to result in irrational beliefs (Andre, 2006; Yang et al., 2007; Samar et al., 2013; Jibeen, 2015). For example, the Big-five personality traits have been proven to predict people's irrational beliefs (Samar et al., 2013; Jibeen, 2015). As important personality characteristics, the Dark Triad traits were closely associated with Big-five personality traits (Lee and Ashton, 2005), thus suggesting that the latter would also predict and affect individuals' irrational and unrealistic beliefs. A series of compelling studies support our inference (Paulhus and Williams, 2002; Lakey et al., 2008; Jones, 2014; Birkas et al., 2015).

Research has shown that high Machiavellianism was associated with greater perceived reward for engaging in deviant behavior and less perceived punishment for that activity (Birkas et al., 2015). This indicates that Machiavellians tend to consider merely the profits they want to pursue, which may result in their erroneous estimation about the odds of rewards or punishment (Rauthmann and Kolar, 2013; Birkas et al., 2015). They may form unrealistic beliefs about good luck, and irrationally overrate the gain-seeking probability and underrate or even neglect the punishment probability. Additionally, individuals with high narcissism often possess a sense of entitlement (Morf et al., 2000) and overconfidence (Campbell et al., 2004), which lead them to misjudge the chances of success. In other words, they may inappropriately raise the subjective probabilities of successes (Paulhus and Williams, 2002). Besides, the inflated self-beliefs caused narcissists to form an unrealistic view that luck always works in their favor, which make them underrate the probability of risks or losses and arrive at irrational decisions (Judge et al., 2006; Chatterjee and Hambrick, 2007; Lakey et al., 2008). As such, people with high narcissism may hold the illusory beliefs that good luck would fall to them and they can control an event. Results from previous research also demonstrated that psychopathy was positively associated with irrational beliefs (Samar et al., 2013); people high in psychopathy tend to exhibit a biased judgment of risk perceptions, or even ignore the inherent risks related to an event (Jones, 2014). In addition, the characteristic of low self-control renders them unable to resist the temptations from unfair advantages, which cause them unable to keep a cool mind to make rational judgments (Tangney et al., 2004). Indeed, all of these would exacerbate the “Dark Triad” people's unrealistic estimations about potential gains or risks. Taken together, it is nature to assume that when faced with deviant behaviors, individuals with high Dark Triad would be more likely to hold irrational beliefs in good luck and make irrational estimations.

#### Belief in good luck and corruption

Ajzen's theory of planned behavior also indicated that individuals' cognitive beliefs about a behavior are considered as the prevailing determinants of their behavioral tendencies (Fishbein and Ajzen, 1975; Ajzen, 1991). Additionally, a number of cognitive-behavioral theories suggested that the deviant behaviors are caused by inaccurate or irrational beliefs (Ellis, 1999; Andre, 2006; Jibeen, 2015). Although, a previous study has suggested that beliefs about luck can serve as a positive expectation for future events to a certain degree (Darke and Freedman, 1997a), when confronted with antisocial and unethical behaviors, such as corruption, this irrational belief will lead to serious consequences. Research has shown that belief in good luck generated the feelings of illusion of control and optimistic bias (Darke and Freedman, 1997a,b), and these unrealistic feelings were prevalent amongst gambling and risk-taking behaviors (Darke and Freedman, 1997b; Chiu and Storm, 2010). For example, the gamblers' perception of themselves being lucky led them to continue gambling (Wohl and Enzle, 2003; Chiu and Storm, 2010); and the lower perceived likelihood of punishment lead people to have a higher perceived corrupt intention (Bai et al., 2014). Therefore, as to corruption, under the influence of lucky belief, irrationally overestimating the likelihood of seeking personal benefits via bribe-offering, and inappropriately underestimating the likelihood of being penalized for bribe-taking, would together make people have a strong tendency to engage in bribe-offering and bribe-taking behaviors. Thus, engaging in corruption is, at least to some degree, dependent upon one's irrational beliefs in good luck in seeking gains or avoiding penalty. We then propose that the more people believe in good luck, the more likely they would be to engage in corruption.

Given that belief in good luck is closely linked to the Dark Triad of personality and corrupt behaviors, based on the theory of planned behavior, it is reasonable to hypothesize that belief in good luck may play a mediating role in the relationship between the Dark Triad traits and corrupt intention (Hypothesis 2).

### Overview of the current studies

Based on the literature review of previous theories and studies, we posited the following two hypotheses:

Hypothesis 1: The Dark Triad traits positively predict corrupt intention; and
Hypothesis 2: Belief in good luck plays a mediating role in the relationship between the Dark Triad traits and corrupt intention.

We conducted two sub-studies in China to test Hypothesis 1 and Hypothesis 2. In these sub-studies, bribe-offering and bribe-taking were used as two different outcome valences of the forms of corruption in hypothetical scenarios, and the measurement of belief in good luck was embedded in the corresponding scenario. In Study 1, we explored the correlations between each Dark Triad trait and bribe-offering intention, and constructed mediation models to verify the assumption that belief in good luck in seeking gains (irrationally overestimating the probability of a positive-valence outcome) would mediate the effect of the Dark Triad traits on bribe-offering intention. In Study 2, we further examined whether each Dark Triad trait could facilitate bribe-taking intention, and tested whether belief in good luck in avoiding penalty (irrationally underestimating the probability of a negative-valence outcome) would mediate the effect of the Dark Triad traits on bribe-taking intention.

## Study 1

The aim of Study 1 was twofold. First, we examined whether the Dark Triad traits could predict bribe-offering intention. We expected that individuals with high Dark Triad traits were more likely engage in bribe-offering behaviors. Second, we explored whether one's belief in good luck can mediate the effects of the Dark Triad traits on bribe-offering intention. We predicted that individuals with Dark Triad tend to engage in corruption partially because they hold the lucky belief and irrationally overestimate the likelihood of seeking gains via bribe-offering.

### Methods

#### Participants

A total of 404 Chinese adults were recruited online, via the Qualtrics Survey from different enterprises in China. The final valid sample comprised 395 Chinese adults (231 female and 164 male; *M*_*age*_ = 29.56 years, *SD* = 6.30 years; age range: 18–60 years), as 9 adults were excluded because 4 of them failed to complete the questionnaires, and 5 responded with extreme values. The effective response rate was 97.77%. Participants varied considerably in terms of their education levels (18.2% with high school education or less, 31.4% with a college degree, 42.0% with a bachelor degree, and 8.4% with a postgraduate degree) and monthly income (14.4% with less than 2000 yuan, 49.1% with 2001–5000 yuan, 24.6% with 5001–8000 yuan, 9.1% with 8001–20,000 yuan, and 2.8% with more than 20,000 yuan).

#### Procedure

After signing a consent form, the participants completed a series of self-report questionnaires within 45 mins. These questionnaires included the Short Dark Triad scale, the bribe-offering intention measure, the belief in good luck measure, the life orientation test revised and the new general self-efficacy scale. After they completed the questionnaires, the participants were asked to provide demographic information, including gender, age, education level and monthly income. Upon completion, participants were thanked and debriefed. Anonymity of the participants was ensured in order to reduce social desirability, and all procedures were approved by the ethics board at the School of Psychology, Beijing Normal University.

#### Measures

##### The short Dark Triad (SD3)

Following Jones and Paulhus (2014) methods, the 27-item Dark Triad scale was translated via a back-translation procedure and then was used to assess the Dark Triad personality traits. This scale is divided into three dimensions: Machiavellianism (e.g., “Make sure your plans benefit yourself, not others”), narcissism (e.g., “People see me as a natural leader”) and psychopathy (e.g., “I like to get revenge on authorities”). Each sub-scale comprises 9 items. Participants were asked to rate their level of agreement with each item on a 5-point Likert scale (1 = *strongly disagree*, 5 = *strongly agree*). Higher scores on the scale indicate higher levels of Dark Triad tendencies. In this study, the Cronbach's α for the total scale was 0.87, and that for Machiavellianism, narcissism and psychopathy was 0.71, 0.78, and 0.78, respectively. A confirmatory factor analysis also showed a good fit for the measurement model (χ^2^ ∕ *df* = 1.83, GFI = 0.91, CFI = 0.90, RMSEA = 0.05). The results of reliability and validity analyses indicated that this scale was applicable to a Chinese sample.

##### The bribe-offering scenario

Participants were asked to read three hypothetical daily life scenarios on bribe-offering, which were generated from a panel discussion (see Supplementary Material). Each participant read the following instructions before the task: “Please vividly imagine that you are in each situation.” The following is a sample of a bribe-offering scenario:

“Suppose you are a section-level employee who has a strong desire to gain a promotion. The municipal government is currently selecting and promoting one section chief. You are in a disadvantaged position in the competition compared with other section-level candidates. Before the final decision, you ask the deputy mayor to help you and plan to privately promise him a certain sum of money as a token of your thanks if you win in the competition. You are aware that winning the competition via offering a bribe is an unlawful behavior.”

After each scenario, propensity to engage in bribe-offering was assessed by instructing the participants to “Please estimate the likelihood you would offer the bribe to someone who is in charge” on a 7-point Likert scale (1 = *extremely unlikely*, 7 = *extremely likely*). The index of bribe-offering intention was calculated as the average score of the three scenarios, where a higher score indicates greater intention of bribe-offering. The Cronbach's α of this tool was 0.80.

##### Belief in good luck

In this study, we adopted the research paradigm of “objective probability event-subjective probability estimation” to measure one's belief in good luck related to corruption. After each bribe-offering scenario, there was a corresponding scenario to measure one's belief in good luck in gain-seeking. The following is a sample of such a scenario related to the previously presented example of a bribe-offering scenario:

“Suppose you have offered a bribe to the deputy mayor privately. According to some recent studies related to this scenario, the probability of securing a promotion via bribe-offering is only about 5% in recent years. Please respond to the following two items: (1) Despite the low probability, however, you still definitely believe that you will have a good luck to gain promotion when you offer the bribe; (2) The good luck makes you believe that your winning probability will be significant higher than 5% when you offer the bribe.”

Participants responded to each item on a 7-point Likert scale (1 = *strongly disagree*, 7 = *strongly agree*). Three scenarios were averaged together to create an indicator of belief in good luck, where higher scores were indicative of higher levels of belief in good luck. The Cronbach's α of this tool was 0.79. It is important to note that according to the statistical probability, the 5% rate that was used in the scenarios present in this study was a very low probability event.

##### Control variables

We included gender, age, education, income, dispositional optimism, and self-efficacy as control variables that potentially influenced the findings. For example, research has suggested that gender, age, education, and income significantly affect corruption (Čábelková and Hanousek, 2004; Donchev and Ujhelyi, 2007). Moreover, optimism and self-efficacy were positively associated with belief in good luck (Darke and Freedman, 1997a; Damisch et al., 2010). Therefore, these variables were assessed and controlled to isolate the independent impacts of the Dark Triad traits and belief in good luck on corrupt intention in our following analyses.

The 10-item Life orientation test revised measure was used to evaluate the participants' level of dispositional optimism (Scheier et al., 1994). Six items were used to assess optimism (e.g. “I'm always optimistic about my future”) and four items used as filler items were not scored. All items were rated on a 5-point Likert scale (1 = *strongly disagree*, 5 = *strongly agree*), with higher scores indicating a greater degree of dispositional optimism. The Cronbach's alpha was 0.83.

Self-efficacy was measured using the 8-item new general self-efficacy scale (Chen et al., 2001). One sample item is “In general, I think that I can obtain outcomes that are important to me”. Participants completed these items on a 5-point Likert scale (1 = *strongly disagree*, 5 = *strongly agree*), with higher scores representing a greater degree of generalized self-efficacy. The Cronbach's alpha was 0.88.

### Results

#### Discriminant validity

To examine the discriminant validity of belief in good luck, we conducted a confirmatory factor analysis on belief in good luck, dispositional optimism, and self-efficacy. Results showed that a three-factor model provided a good fit to the data [$\chi_{\left(116,\;\text{N}\;=\;395\right)}^{2}$ = 278.45, *p* < 0.001, GFI = 0.92, CFI = 0.93, RMSEA = 0.06], all factor loadings were statistically significant, with standardized loadings ranging from 0.60 to 0.80. Model fit was significantly better for the three-factor model compared with a single-factor model [$\Delta \chi_{\left(3,\;\text{N}\;=\;395\right)}^{2}$ = 998.50, *p* < 0.001], a two-factor model that combined belief in good luck and dispositional optimism into one factor [$\Delta \chi_{\left(2,\;\text{N}\;=\;395\right)}^{2}$ = 336.43, *p* < 0.001], and a two-factor model that combined belief in good luck and self-efficacy into one factor [$\Delta \chi_{\left(2,\;\text{N}\;=\;395\right)}^{2}$ = 343.23, *p* < 0.001].

#### Descriptive analyses

Means, standard deviations, and zero-order correlation coefficients among the variables have been presented in Table 1. As hypothesized, the results showed that there were significant correlations between each Dark Triad trait, belief in good luck, and bribe-offering intention. More specifically, Machiavellianism, narcissism, and psychopathy were positively correlated with both belief in good luck and bribe-offering intention.

**Table 1:** **Descriptive statistics and correlations among the variables**.

**Variables**	***M***	***SD***	**1**	**2**	**3**	**4**	**5**	**6**	**7**	**8**	**9**	**10**	**11**
1. Gender	0.58	0.49	−										
2. Age	29.56	6.30	−0.02	−									
3. Education	2.41	0.88	0.05	−0.10^*^	−								
4. Income	2.37	0.93	−0.13^*^	0.12^*^	0.27^***^	−							
5. Optimism	3.37	0.66	−0.02	−0.04	0.05	0.004	−						
6. Self-efficacy	3.54	0.65	−0.12^*^	0.12^*^	0.03	0.07	0.13^**^	−					
7. Machiavellianism	3.19	0.58	−0.13^*^	−0.10	0.04	0.05	0.13^*^	0.12^*^	−				
8. Narcissism	2.80	0.66	−0.18^***^	−0.19^***^	0.11^*^	0.08	0.14^**^	0.16^**^	0.46^***^	−			
9. Psychopathy	2.42	0.65	−0.18^***^	−0.14^**^	0.003	−0.02	0.12^*^	0.07	0.34^***^	0.63^***^	−		
10. BIGL	4.14	1.24	−0.16^**^	0.04	0.06	0.10^*^	0.12^*^	0.10^*^	0.26^***^	0.33^***^	0.17^**^	−	
11. BOI	3.90	1.45	−0.13^*^	−0.07	0.04	0.07	0.16^**^	0.10	0.34^***^	0.20^***^	0.19^***^	0.33^***^	−

BIGL, Belief in good luck; BOI, Bribe-offering intention. Gender was dummy coded as 0 = male and 1 = female. Education was coded as 1 = high school education or less, 2 = college degree, 3 = bachelor degree and 4 = postgraduate degree. Monthly income (CNY) was coded as 1 = under 2000, 2 = 2001–5000, 3 = 5001–8000, 4 = 8001–20,000 and 5 = above 20,000.

* p < 0.05;

** p < 0.01;

*** p < 0.001.

#### Testing the mediating role of belief in good luck in the relationship between the Dark Triad traits and bribe-offering intention

In order to test Hypothesis 1 that the Dark Triad traits would predict corrupt intention, we first entered the control variables and then the three Dark Triad traits in the hierarchical regression analysis. The results showed that Machiavellianism (β = 0.30, *p* < 0.001, 95% CI [0.22, 0.39]), narcissism (β = 0.15, *p* < 0.01, 95% CI [0.04, 0.25]), and psychopathy (β = 0.15, *p* < 0.01, 95% CI [0.05, 0.25]) were positive predictors of bribe-offering intention. Thus, Hypothesis 1 was supported, indicating that people with higher levels of the Dark Triad tendencies were likely to exhibit a higher corrupt intention.

**Figure 1:**
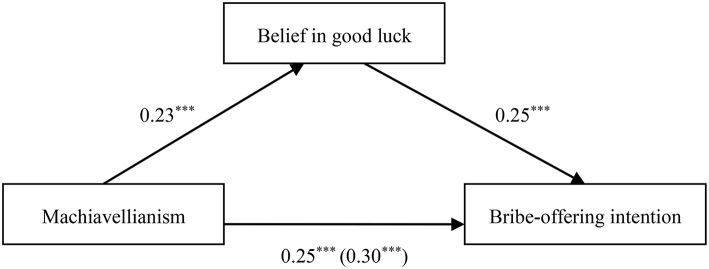
**Indirect effect of belief in good luck on the link between Machiavellianism and bribe-offering intention**. ***p < 0.001.

**Figure 2:**
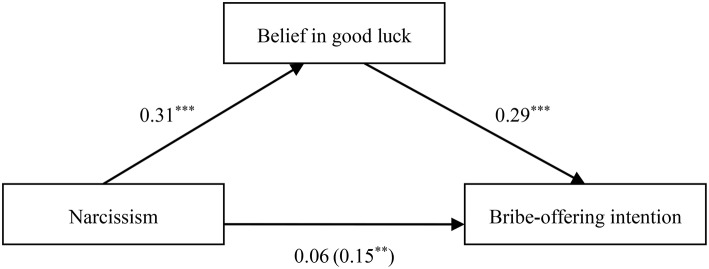
**Indirect effect of belief in good luck on the link between narcissism and bribe-offering intention**. **p < 0.01; ***p < 0.001.

**Figure 3:**
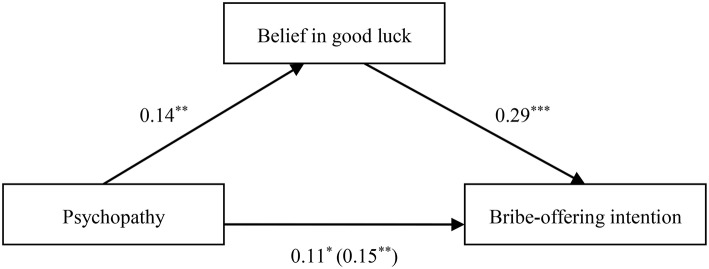
**Indirect effect of belief in good luck on the link between psychopathy and bribe-offering intention**. *p < 0.05; **p < 0.01; ***p < 0.001.

To explain the psychological process underlying the effects of the Dark Triad traits on corrupt intention, we conducted regression analyses according to the specification set out by Andrew Hayes' (2013) PROCESS for SPSS using Model 4 (a bootstrapping CI method with *N* = 5000 bootstrap samples) to test the mediation effect of belief in good luck on the relationship between the Dark Triad traits and corrupt intention. The mediation effects were statistically significant when the 95% confidence intervals did not include zero. To yield standardized coefficients, all variables were converted to *z*-scores prior to analysis. As illustrated in Tables 2–**4** and Figures 1–**3**, after adjusting for the control variables, belief in good luck in gain-seeking was found to mediate the associations between each Dark Triad trait and bribe-offering intention. Thus, Hypothesis 2 was confirmed.

**Table 2:** **Test the mediation effect of Belief in good luck on the link between Machiavellianism and Bribe-offering intention (*N* = 395)**.

**Predictors**	**Belief in good luck**			**Bribe-offering intention**		
	**β**	***t***	**95%CI**	**β**	***t***	**95%CI**
Gender	−0.12^*^	−2.48	[−0.22, −0.03]	−0.04	−0.95	[−0.14, 0.05]
Age	0.06	1.15	[−0.04, 0.15]	−0.06	−1.30	[−0.15, 0.03]
Education	0.05	0.90	[−0.05, 0.15]	−0.01	−0.11	[−0.10, 0.09]
Income	0.05	1.06	[−0.05, 0.15]	0.03	0.59	[−0.07, 0.12]
Optimism	0.09	1.74	[−0.01, 0.18]	0.09	1.87	[−0.005, 0.18]
Self-efficacy	0.04	0.72	[−0.06, 0.13]	0.03	0.69	[−0.06, 0.13]
Machiavellianism	0.23^***^	4.60	[0.13, 0.33]	0.25^***^	5.11	[0.15, 0.34]
Belief in good luck				0.25^***^	5.16	[0.15, 0.34]
*R^2^*		0.10			0.20	
*F*		6.39^***^			11.69^***^	

Each column set is a regression equation that predicts the criterion at the top of the column.

* p < 0.05;

*** p < 0.001.

When Machiavellianism was the independent variable, the link between Machiavellianism and bribe-offering intention was significantly mediated by belief in good luck in seeking gains (β_*indirect*_ = 0.06, *SE* = 0.02, 95% CI [0.03, 0.10]), as depicted in Table 2 and Figure 1.

When narcissism was the independent variable, we found that the relationship between narcissism and bribe-offering intention was fully mediated by belief in good luck in seeking gains (β_*indirect*_ = 0.09, *SE* = 0.02, 95% CI [0.05, 0.14]), as depicted in Table 3 and Figure 2.

**Table 3:** **Test the mediation effect of Belief in good luck on the link between Narcissism and Bribe-offering intention (*N* = 395)**.

**Predictors**	**Belief in good luck**			**Bribe-offering intention**		
	**β**	***t***	**95%CI**	**β**	***t***	**95%CI**
Gender	−0.10^*^	−2.03	[−0.19, −0.003]	−0.06	−1.18	[−0.15, 0.04]
Age	0.10	1.94	[−0.002, 0.19]	−0.08	−1.57	[−0.18, 0.02]
Education	0.03	0.56	[−0.07, 0.13]	−0.01	−0.13	[−0.10, 0.09]
Income	0.05	0.90	[−0.05, 0.14]	0.03	0.63	[−0.07, 0.13]
Optimism	0.08	1.60	[−0.02, 0.17]	0.10^*^	2.15	[0.01, 0.20]
Self-efficacy	0.02	0.30	[−0.08, 0.11]	0.05	0.96	[−0.05, 0.14]
Narcissism	0.31^***^	6.16	[0.21, 0.41]	0.06	1.09	[−0.05, 0.16]
Belief in good luck				0.29^***^	5.69	[0.19, 0.39]
*R*^2^		0.14			0.14	
*F*		8.92^***^			8.05^***^	

Each column set is a regression equation that predicts the criterion at the top of the column.

* p < 0.05;

*** p < 0.001.

When psychopathy was the independent variable, the results indicated that belief in good luck in seeking gains partially mediated the relationship between psychopathy and bribe-offering intention (β_*indirect*_ = 0.04, *SE* = 0.02, 95% CI [0.01, 0.08]), as depicted in Table 4 and Figure 3.

**Table 4:** **Test the mediation effect of Belief in good luck on the link between Psychopathy and Bribe-offering intention (*N* = 395)**.

**Predictors**	**Belief in good luck**			**Bribe-offering intention**		
	**β**	***t***	**95%CI**	**β**	***t***	**95%CI**
Gender	−0.12^*^	−2.41	[−0.22, −0.02]	−0.05	−0.97	[−0.14, 0.05]
Age	0.05	1.04	[−0.05, 0.15]	−0.07	−1.53	[−0.17, 0.02]
Education	0.05	0.97	[−0.05, 0.15]	−0.002	−0.05	[−0.10, 0.10]
Income	0.07	1.26	[−0.04, 0.17]	0.04	0.75	[−0.06, 0.14]
Optimism	0.10	1.91	[−0.003, 0.19]	0.10^*^	2.06	[0.004, 0.19]
Self-efficacy	0.05	1.02	[−0.05, 0.15]	0.05	1.02	[−0.05, 0.14]
Psychopathy	0.14^**^	2.85	[0.04, 0.24]	0.11^*^	2.15	[0.01, 0.20]
Belief in good luck				0.29^***^	5.95	[0.19, 0.39]
*R*^2^		0.07			0.15	
*F*		4.41^***^			8.56^***^	

Each column set is a regression equation that predicts the criterion at the top of the column.

* p < 0.05;

** p < 0.01;

*** p < 0.001.

### Discussion

The results supported Hypothesis 1 and 2, demonstrated that the Dark Triad traits foster corruption and this effect was mediated by belief in good luck. The association between narcissism and bribe-offering intention was fully mediated by belief in good luck, while the effects of Machiavellianism and psychopathy on bribe-offering intention were both partially mediated by belief in good luck. Individuals with Dark Triad tended to be driven by personal goals and interests (Jonason and Webster, 2012), even at a cost to other people. They held good luck beliefs and overestimated their chances of seeking unfair advantages via bribe-offering, and may judge that one cannot succeed in the competition without bribe-offering and that the benefit of winning the competition clearly outweighs the cost of bribe-offering.

Study 1 mainly focused on one mechanism related to belief in good luck, that is, people with the Dark Triad traits tended to overestimate the probability of positive-valence outcomes. However, it was unclear how they would react when faced with the negative-valence outcomes of corruption, such as penalty. To further explore the mediating role of belief in good luck, we conducted Study 2.

## Study 2

In Study 2, we used a different context and sample to examine the association between each of the Dark Triad traits and bribe-taking intention, and examined the mediating role of belief in good luck in this relationship. We speculated that individuals with Dark Triad tend to engage in bribe-taking behaviors partially because they hold the lucky belief and irrationally underestimate the likelihood of being penalized for bribe-taking.

### Methods

#### Participants

A total of 386 Chinese adults were recruited online, via the Qualtrics Survey from different enterprises in China. The final valid sample size was 382 Chinese adults (193 female and 189 male; *M*_*age*_ = 28.19 years, *SD* = 5.66 years; age range: 18–63 years), as 4 adults were excluded because they failed to complete the questionnaires. The effective response rate was 98.96%. Participants varied considerably in terms of their education levels (12.8% with high school education or less, 39.3% with a college degree, 43.5% with a bachelor degree, and 4.5% with a postgraduate degree) and monthly income (8.4% with less than 2000 yuan, 48.4% with 2001–5000 yuan, 30.9% with 5001–8000 yuan, 9.4% with 8001–20,000 yuan, and 2.9% with more than 20,000 yuan).

#### Procedure

The study procedure was the same as that employed in Study 1.

#### Measures

##### The short Dark Triad (SD3)

The 27-item Dark Triad was used, as in Study 1. In this study, the Cronbach's α for the total scale was 0.87, and that for Machiavellianism, narcissism, and psychopathy was 0.78, 0.81, and 0.77, respectively.

##### The bribe-taking scenario

We adapted bribery scenarios successfully used in past research (Bai et al., 2014). Participants were exposed to three hypothetical daily life scenarios about bribe-taking (see Supplementary Material). The experimental procedure was the same as that employed in Study 1. The following is a sample of the bribe-taking scenario:

“Suppose you are a director who is in charge of bidding. Compared to other bidders, Company A is in an unfavorable position in the competition. In order to win the bid, the CEO of Company A asks you to help him, and also privately promises you a certain sum of money if his company wins the bid. If you help him, the probability that he will win the bid will be greatly improved. But you are aware that it is against the law to help him win the bid by accepting a bribe.”

After each scenario, propensity to engage in bribe-taking was measured by “Please estimate the likelihood that you would offer the help to Company A” on a 7-point Likert scale (1 = *extremely unlikely*, 7 = *extremely likely*). The index of bribe-taking intention was calculated as the average score of the three scenarios, where higher scores are indicative of greater bribe-taking intention. The Cronbach's α of the tool was 0.85.

##### Belief in good luck

The research paradigm of “objective probability event-subjective probability estimation” was used, same as in Study 1. After each bribe-taking scenario, there was a corresponding scenario to measure one's belief in good luck in penalty-avoidance. The following is a sample scenario that corresponds to the example illustrated in the bribe-taking scenario:

“Suppose you have accepted the bribe that the CEO of Company A offered to you and helped him to win the bid. According to the statistics of the national department related to this scenario, the probability of penalty for bribe-taking in bidding is almost 40% in recent years. Please respond to the following two items: (1) Despite the high probability, however, you still definitely believe that you will have a good luck to avoid penalty; (2) The good luck makes you believe that your probability of being penalized will be significant lower than 40% even if you take the bribe.”

Participants responded to each item on a 7-point Likert scale (1 = *strongly disagree*, 7 = *strongly agree*). The average of three scenarios comprised the score of belief in good luck. The higher the averaged score, the higher is the level of belief in good luck. The Cronbach's α of this tool was 0.89. Additionally, we should note that though China is currently ramping up efforts to fight corruption, the results of a pilot investigation show that the penalty rate in corruption cases is just about 10%, and the 40% rate that was presented in the scenarios used in this study is a very high penalty rate.

##### Control variables

We controlled the same variables as in Study 1. The Cronbach's α of the 10-item Life orientation test revised measure and the new general self-efficacy scale was 0.81, and 0.88, respectively.

### Results

#### Discriminate validity

The procedure to test the discriminant validity of belief in good luck was the same as that employed in Study 1. Results demonstrated that the three-factor model was better fit the data [χ^2^_(116, **N** = 382)_ = 335.88, *p* < 0.001, GFI = 0.90, CFI = 0.92, RMSEA = 0.07] than the single-factor model [χ^2^_(3, **N** = 382)_ = 1188.22, *p* < 0.001], the two-factor model that combined belief in good luck and dispositional optimism into one factor [Δχ^2^_(2, **N** = 382)_ = 636.56, *p* < 0.001], and the two-factor model that combined belief in good luck and self-efficacy into one factor [Δχ^2^_(2, **N** = 395)_ = 645.50, *p* < 0.001].

#### Descriptive statistics

Table 5 displays the descriptive statistics and zero-order correlation coefficients among the variables. As expected, narcissism, and psychopathy were positively correlated with belief in good luck and bribe-taking intention. Interestingly, Machiavellianism was positively related to bribe-taking intention, but was not significantly linked to the corresponding belief in good luck.

**Table 5:** **Descriptive statistics and correlations among the variables**.

**Variables**	***M***	***SD***	**1**	**2**	**3**	**4**	**5**	**6**	**7**	**8**	**9**	**10**	**11**
1. Gender	0.51	0.50	−										
2. Age	28.19	5.66	−0.04	−									
3. Education	2.40	0.77	−0.12^*^	0.05	−								
4. Income	2.50	0.88	−0.27^***^	0.04	0.30^***^	−							
5. Optimism	3.36	0.73	−0.04	−0.04	0.07	−0.06	−						
6. Self-efficacy	3.51	0.69	−0.04	0.05	0.05	0.12^*^	0.11^*^	−					
7. Machiavellianism	3.20	0.67	−0.17^**^	−0.08	0.11^*^	0.02	0.14^**^	0.14^**^	−				
8. Narcissism	2.90	0.69	−0.20^***^	−0.05	0.16^**^	0.07	0.13^**^	0.14^**^	0.44^***^	−			
9. Psychopathy	2.46	0.67	−0.18^***^	−0.11^*^	0.11^*^	0.04	0.15^**^	0.04	0.37^***^	0.54^***^	−		
10. BIGL	3.56	1.72	−0.18^***^	0.05	0.11^*^	0.13^*^	0.12^*^	0.10^*^	0.09	0.26^***^	0.18^***^	−	
11. BTI	3.09	1.56	−0.18^***^	−0.18^**^	0.01	0.08	0.14^**^	0.04	0.28^***^	0.18^***^	0.30^***^	0.32^***^	−

BIGL, Belief in good luck; BTI, Bribe-taking intention. Gender was dummy coded as 0 = male and 1 = female. Education was coded as 1 = high school education or less, 2 = college degree, 3 = bachelor degree and 4 = postgraduate degree. Monthly income (CNY) was coded as 1 = under 2000; 2 = 2001–5000; 3 = 5001–8000; 4 = 8001–20,000; and 5 = above 20,000.

* p < 0.05;

** p < 0.01;

*** p < 0.001.

#### Testing the mediating role of belief in good luck in the relationship between the Dark Triad traits and bribe-taking intention

While controlling the control variables, Machiavellianism (β = 0.24, *p* < 0.001, 95% CI [0.15, 0.34]), narcissism (β = 0.13, *p* < 0.05, 95% CI [0.03, 0.25]), and psychopathy (β = 0.25, *p* < 0.001, 95% CI [0.15, 0.34]) was positively predicted bribe-taking intention. Thus, Hypothesis 1 was verified again.

**Figure 4:**
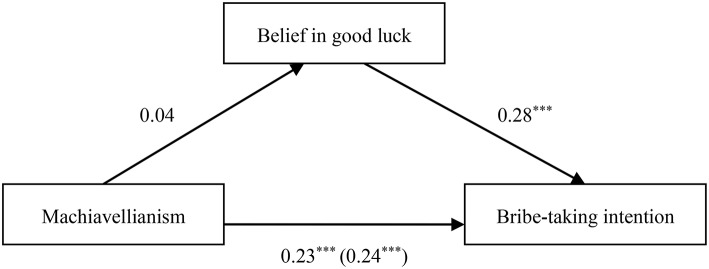
**Indirect effect of belief in good luck on the link between Machiavellianism and bribe-taking intention**. ***p < 0.001.

**Figure 5:**
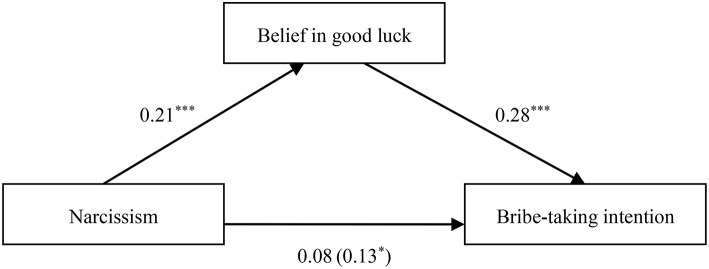
**Indirect effect of belief in good luck on the link between narcissism and bribe-taking intention**. *p < 0.05; ***p < 0.001.

**Figure 6:**
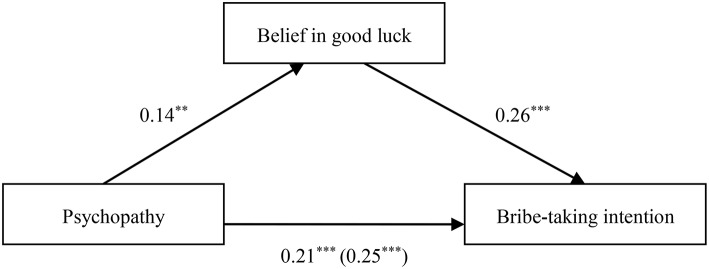
**Indirect effect of belief in good luck on the link between psychopathy and bribe-taking intention**. **p < 0.05; ***p < 0.001.

We then examined whether belief in good luck in penalty-avoidance mediated the effect of each Dark Triad trait on bribe-taking intention. Similar to that in Study 1, the Model 4 of the Hayes' PROCESS macro for SPSS was adopted (Hayes, 2013). The results have been illustrated in Tables 6–**8** and Figures 4–**6**.

**Table 6:** **Test the mediation effect of Belief in good luck on the link between Machiavellianism and Bribe-taking intention (*N* = 382)**.

**Predictors**	**Belief in good luck**			**Bribe-taking intention**		
	**β**	***t***	**95%CI**	**β**	***t***	**95%CI**
Gender	−0.14^**^	−2.63	[−0.24, −0.03]	−0.09	−1.89	[−0.19, 0.003]
Age	0.04	0.82	[−0.06, 0.14]	−0.17^***^	−3.61	[−0.26, −0.08]
Education	0.06	1.06	[−0.05, 0.16]	−0.07	−1.51	[−0.17, 0.02]
Income	0.07	1.21	[−0.04, 0.17]	0.05	1.03	[−0.05, 0.15]
Optimism	0.10^*^	2.01	[0.002, 0.20]	0.07	1.49	[−0.02, 0.16]
Self-efficacy	0.07	1.32	[−0.03, 0.17]	−0.02	−0.50	[−0.12, 0.07]
Machiavellianism	0.04	0.80	[−0.06, 0.14]	0.23^***^	4.82	[0.14, 0.32]
Belief in good luck				0.28^***^	5.93	[0.19, 0.37]
*R*^2^		0.06			0.21	
*F*		3.67^***^			12.62^***^	

Each column set is a regression equation that predicts the criterion at the top of the column.

* p < 0.05;

** p < 0.01;

*** p < 0.001.

When Machiavellianism was the independent variable, the results showed that belief in good luck in penalty-avoidance did not mediate the relationship between Machiavellianism and bribe-taking intention (β_*indirect*_ = 0.01, *SE* = 0.02, 95% CI [−0.02, 0.05]), as depicted in Table 6 and Figure 4.

When narcissism was the independent variable, the relationship between narcissism and bribe-taking intention was fully mediated by belief in good luck in penalty-avoidance (β_*indirect*_ = 0.06, *SE* = 0.02, 95% CI [0.03, 0.11]), as depicted in Table 7 and Figure 5.

**Table 7:** **Test the mediation effect of Belief in good luck on the link between Narcissism and Bribe-taking intention (*N* = 382)**.

**Predictors**	**Belief in good luck**			**Bribe-taking intention**		
	**β**	***t***	**95%CI**	**β**	***t***	**95%CI**
Gender	−0.11^*^	−2.03	[−0.21, −0.004]	−0.12^*^	−2.33	[−0.22, −0.02]
Age	0.05	1.03	[−0.05, 0.15]	−0.18^***^	−3.84	[−0.28, −0.09]
Education	0.03	0.62	[−0.07, 0.13]	−0.06	−1.18	[−0.16, 0.04]
Income	0.07	1.29	[−0.04, 0.17]	0.04	0.79	[−0.06, 0.14]
Optimism	0.09	1.72	[−0.01, 0.18]	0.09	1.80	[−0.01, 0.18]
Self-efficacy	0.05	0.95	[−0.05, 0.15]	−0.002	−0.05	[−0.10, 0.09]
Narcissism	0.21^***^	4.16	[0.11, 0.31]	0.08	1.48	[−0.02, 0.17]
Belief in good luck				0.28^***^	5.52	[0.18, 0.37]
*R*^2^		0.10			0.17	
*F*		6.21^***^			9.48^***^	

Each column set is a regression equation that predicts the criterion at the top of the column.

* p < 0.05;

*** p < 0.001.

When psychopathy was the independent variable, the results demonstrated that belief in good luck in penalty-avoidance partially mediated the relationship between psychopathy and bribe-taking intention (β_*indirect*_ = 0.04, *SE* = 0.02, 95% CI [0.01, 0.07]), as depicted in Table 8 and Figure 6.

**Table 8:** **Test the mediation effect of Belief in good luck on the link between Psychopathy and Bribe-taking intention (*N* = 382)**.

**Predictors**	**Belief in good luck**			**Bribe-taking intention**		
	**β**	***t***	**95%CI**	**β**	***t***	**95%CI**
Gender	−0.12^*^	−2.31	[−0.22, −0.02]	−0.10	−1.95	[−0.19, 0.001]
Age	0.05	1.07	[−0.04, 0.15]	−0.16^***^	−3.46	[−0.25, −0.07]
Education	0.05	0.90	[−0.06, 0.15]	−0.07	−1.39	[−0.16, 0.03]
Income	0.07	1.24	[−0.04, 0.17]	0.04	0.88	[−0.06, 0.14]
Optimism	0.09	1.77	[−0.01, 0.19]	0.07	1.47	[−0.02, 0.16]
Self-efficacy	0.07	1.38	[−0.03, 0.17]	0.002	0.97	[−0.09, 0.09]
Psychopathy	0.14^**^	2.65	[0.04, 0.24]	0.21^***^	4.33	[0.11, 0.31]
Belief in good luck				0.26^***^	5.45	[0.17, 0.36]
*R^2^*		0.08			0.20	
*F*		4.64^***^			11.95^***^	

Each column set is a regression equation that predicts the criterion at the top of the column.

* p < 0.05;

** p < 0.01;

*** p < 0.001.

### Discussion

These results reconfirmed Hypothesis 1 and showed that the Dark Triad traits were positively associated with corruption. Additionally, Hypothesis 2 was partially verified. The effect of narcissism on bribe-taking intention was fully mediated by belief in good luck, whereas the effect of psychopathy was partially mediated by belief in good luck. This indicates that “dark” individuals' exaggerated beliefs in good luck may engender a false sense of control (Darke and Freedman, 1997a,b), which may cause them to ignore the real risks of bribe-taking and underestimate the odds of an unfavorable consequence. Interestingly, however, belief in good luck in penalty-avoidance did not mediate the relationship between Machiavellianism and bribe-taking intention. The calculated strategy of Machiavellianism may help us explain this result.

## General discussion

It is important to note that very few studies have examined the mediating role of irrational beliefs in good luck between the Dark Triad of personality and corruption. Our results indicated that the three Dark Triad traits significantly contributed to explaining the variance in corrupt intention. More importantly, the mediating effects suggest that people with Dark Triad are more likely to engage in corruption, partially due to their belief in good luck. In other words, they tend to overestimate the likelihood of seeking gains via bribe-offering and underestimate the likelihood of being penalty for bribe-taking irrationally.

Consistent with the theory of planned behavior (Fishbein and Ajzen, 1975; Ajzen, 1991), the Dark Triad of personality predicted the irrational behavioral beliefs in good luck, which in turn, affected one's corrupt intention. While the Chinese central government has begun prioritizing anti-corruption work and has drastically intensified anti-corruption campaigns, engagement in corruption, especially bribe-taking, has become increasingly risky (Gong, 2002). In Chinese legal sanctions of corruption, engaging in bribe-taking will be penalized severely, whereas bribe-offering may be punished leniently or may even be exempted from investigation (Lu and Gunnison, 2003; Wang and Wu, 2012). Additionally, in the daily social norms of China, people often adopt double standards toward bribe-taking and bribe-offering behaviors. For example, after bribery cases are exposed, people often express their condemnation toward bribe recipients, but show less negativity toward bribe payers. Accordingly, in Study 1, in order to gain an unfair advantage, people with a dark personality were found to tend to overestimate the probability of seeking gains via bribe-offering, which led them to have a tendency to engage in corruption. In Study 2, when faced with a severe penalty for bribe-taking in China, people with high narcissism and psychopathy tended to underestimate or even ignore the penalty odds, which drove them to engage in corruption. However, the strategic nature of Machiavellians protects them from generating an irrational belief in good luck in avoiding penalty, such that they only accept bribes when minimal or no threat of penalty exists.

In line with Hypothesis 1, Machiavellianism positively predicts corruption. Machiavellians employ manipulative, exploitive, and devious methods to achieve private goals and make unethical choices if chances for benefit emerge (Gunnthorsdottir et al., 2002; Birkas et al., 2015). However, one inconsistency in the results is that, in Study 1, Machiavellianism was positively related to belief in good luck in seeking gains, whereas, in Study 2, Machiavellianism was less correlated with belief in good luck in avoiding penalty. In hindsight, these results appear to be consistent with previous theories presented in the Machiavellianism literature. Specifically, recent empirical evidence has shown that Machiavellians were sensitive to rewards (Birkas et al., 2015), likely making reward-oriented decisions, and thus overrating the benefits and probability of gaining an unfair advantage derived from bribe-offering, positively linking to their irrational belief in good luck, which, in part, fuels their tendency to engage in corruption. Perhaps, not surprisingly, Machiavellians only accept bribes when there is maximal benefit with minimal punishment (Jones, 2013). That is, they strategize to maximize their long-term gains (Jones, 2013), and only involve in some cautious misbehaviors. However, since bribe-taking behavior attracts a severe legal penalty in China (Lu and Gunnison, 2003), it may bring in minor benefits in a short period of time but at the expense of significant costs in the long run. With stronger detecting and evaluating abilities, Machiavellians will carefully estimate the potential risk to their own interests (Birkas et al., 2015), and thus may not engender the irrational beliefs in good luck in avoiding penalty. These results are exactly in line with previous evidence that Machiavellianism is associated with anti-social behaviors only when there is no or little risk of being caught (Jones, 2013). Accordingly, Machiavellianism has little correlation with an irrational belief in underestimating the probability of penalty for bribe-taking. These findings about Machiavellianism may be an interesting area for future research.

Obviously, it is reasonable to argue that individuals with high narcissism tend to engage in corruption because they believe in good luck. Our results are in accordance with previous research that narcissists who believe in good luck are overconfident (Darke and Freedman, 1997b; Jones, 2013), which causes them to exhibit cognitive biases in success or penalty perceptions about corruption (Chatterjee and Hambrick, 2007; Lakey et al., 2008). In addition, narcissists possess an overly positive self-concept (Lakey et al., 2008), leading them to acquire a control illusion such that they believe that they could control their corrupt actions (Farwell and Wohlwend-Lloyd, 1998; Jones, 2013). This would further exacerbate their irrational beliefs in good luck. Research has shown that perceived behavioral control influence one's corrupt intention (Rabl and Kuhlmann, 2008). If narcissists believe that they will have a good luck and can control the whole corrupt event, they would self-aggrandize the likelihood of success and downplay the likelihood of penalty. Even when faced with opposite facts, it seems that they still hold the illusory belief that things will go as they wish. Thus, in support of our Hypothesis 2, narcissists hold good luck belief and have a tendency to overestimate their chances of winning via bribe-offering, even though the chance is very low; and underestimate the probability of being penalized for bribe-taking, even though the odds are very high; which would drive them to engage in corrupt behaviors.

Additionally, as predicted, people with high psychopathy tend to engage in corruption. These results furnished preliminary evidence that the erratic antisocial and reckless nature of psychopathy easily lead them to engage in corrupt behaviors (Jones, 2013). Psychopathic individuals with lower self-control (Tangney et al., 2004) cannot resist the temptation of corruption. Lured by potential gains, such individuals seem to be willing to involve in corruption. In addition, mediational data demonstrate that the effect of psychopathy on corrupt intention is partially explained by an irrational belief in good luck. Individuals high in psychopathy cannot regulate impulses effectively and easily create irrational beliefs in good luck in seeking gains or avoiding penalty, and even view gains or penalty as merely a by-product of corruption. These findings confirm our hypothesis that individuals' psychopathy can positively influence their irrational beliefs in good luck, which, in turn, partially leads to a higher corrupt intention.

### Implications

Our research brings significant theoretical implications for the literature on corruption. To our knowledge, this study is among the first attempts to examine the impact of the Dark Triad traits on corruption and on the mediating role of belief in good luck. Firstly, this study extends the preliminary research on corruption from the perspective of individual differences and confirms the relationship between each Dark Triad trait and corruption. Rampant corruption events have raised questions surrounding the personality traits responsible for corruption. In other words, do certain personality traits facilitate corruption? To a certain extent, the current study seems to have answered this question by revealing that people with high Dark Triad of personality are more easily engage in corruption. Secondly, the present study furthered the research on the theory of planned behavior, and encourages researchers to understand the occurrence of corruption by providing insight into the underlying psychological mechanisms between the Dark Triad of personality and corruption. The mediating role of belief in good luck helps reveal the reason why the Dark Triad traits facilitate corruption. Thirdly, these findings also enrich the studies on the prospect theory and establish that people's decisions about the negative or deviant events are not independent of outcome valences once the probabilities are specified. In the present study, we redefined the concept of belief in good luck by using an adapted research paradigm of “objective probability event-subjective probability estimation,” and confirmed its two mechanisms by examining the two different outcome valences of corruption forms.

It is noteworthy that this study was also pragmatic because it provided some anti-corruption measures. First, at the individual level, although one's personality cannot be easily changed, if individuals could be aware that their personality predisposes them to generate unrealistic beliefs in good luck and to engage in corrupt behaviors, then they could take more positive steps to deter them. Second, at the government level, the knowledge that the Dark Triad and irrational beliefs in good luck are associated with corrupt intention can help anti-corruption agencies and institutions become more effective in their actions of restraining corruption. In view of the mediating role of luck beliefs, anti-corruption policies should focus on inhabiting people's irrational beliefs in corruption. For those with high Dark Triad tendencies, the government can decrease their corrupt behaviors by discouraging their irrational belief in good luck in corruption through some ways. For example, creating a fair competition environment is the permanent solution to reduce the necessity of offering bribe and to decrease the probability of gaining profits via bribe-offering. This will encourage people to win competitions or seek benefits through appropriate means. In addition, extensively exposing people to the information about anti-corruption policies (Song and Cheng, 2012), such as the severe penalty policy, can help them be acutely aware of the huge cost of engaging in corruption. That is, both bribe recipients and payers should be penalized heavily. Therefore, stepping up penalties for bribe-offering is also imperative for curbing corruption in China.

### Limitations and prospects

There is no doubt that this study has several limitations. First of all, the cross-sectional data and correlational design does not allow us to detect the causal link between the Dark Triad of personality, belief in good luck, and corruption. Longitudinal studies should be conducted to replicate these findings in future. Second, it is also important to note that although self-report measures are widely used and the instruments employed in present study have good reliability, a response bias and common-method bias are still inevitable. Third, measures of corruption were based on the hypothetical scenarios, which may not reflect actual corrupt behaviors. Thus, the ecological validity of the assessment may be affected. Future research can develop alternative tools to bring research results closer to actual behaviors. Fourth, we should point out that our conclusions are based on the mediation model that we examined with each of the three Dark Triad traits as a predictor variable, belief in good luck as a mediator variable, and corrupt intention as a dependent variable. Although, our results supported the causal processes proposed in the hypothesis development sections, this study did not test the competing models, and therefore, the alternative models and alternative mediators (e.g., moral disengagement) need to be identified in future studies. Finally, the moderator variables between the Dark Triad of personality and corruption also need to be investigated to uncover the boundary conditions, which may help us understand the extent to which the Dark Triad of personality increases corruption.

## Conclusions

This study contributes to the emerging literature concerning the occurrence of corruption from the perspective of individual factors, and the findings hold substantive implications, both theoretical and practical. Using some hypothetical scenarios of corruption in the Chinese context, the two sub-studies in the current study not only present evidence that people with high Dark Triad tendencies are more likely to engage in corruption, but also support the role of their irrational beliefs in good luck as a mediator in this association. We hope that this study can provide some new insights and offer a valuable foundation for the future research on corruption.

## Author contributions

To conception and design: HZ, HZ, and YX. Collection, analysis and interpretation of data: HZ, HZ. Drafting the article: HZ. Revising the article critically: HZ, HZ, and YX.

### Conflict of interest statement

The authors declare that the research was conducted in the absence of any commercial or financial relationships that could be construed as a potential conflict of interest.
