# Supplementary material for: Does the Dark Triad of Personality Predict Corrupt Intention? The Mediating Role of Belief in Good Luck
Source: Front Psychol. 2016 Apr 28;7:608. doi: 10.3389/fpsyg.2016.00608 (PMC4848443; doi:10.3389/fpsyg.2016.00608)

Note: The index of belief in good luck in seeking gains, bribe-offering intention, belief in good luck in avoiding penalty, and bribe-taking intention was calculated as the average score of the three corresponding scenarios respectively, therefore, the numbers, such as 1.33, 1.67, 2.33, and so on were appear in Figures as follows:

★ Study 1:

The data distribution of belief in good luck in seeking gains (*M_{ean}* = 4.14, *M_{edian}* = 4.00, *M_{ode}* = 4.00, *SD* = 1.24):

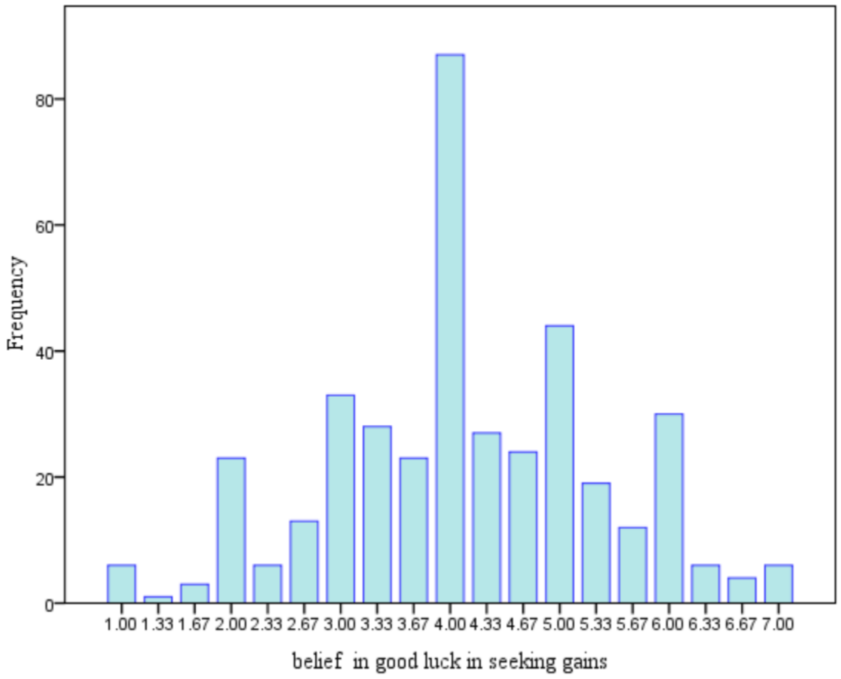

The data distribution of bribe-offering intention (*M_{ean}* = 3.90, *M_{edian}* = 4.00, *M_{ode}* = 4.00, *SD* = 1.45):

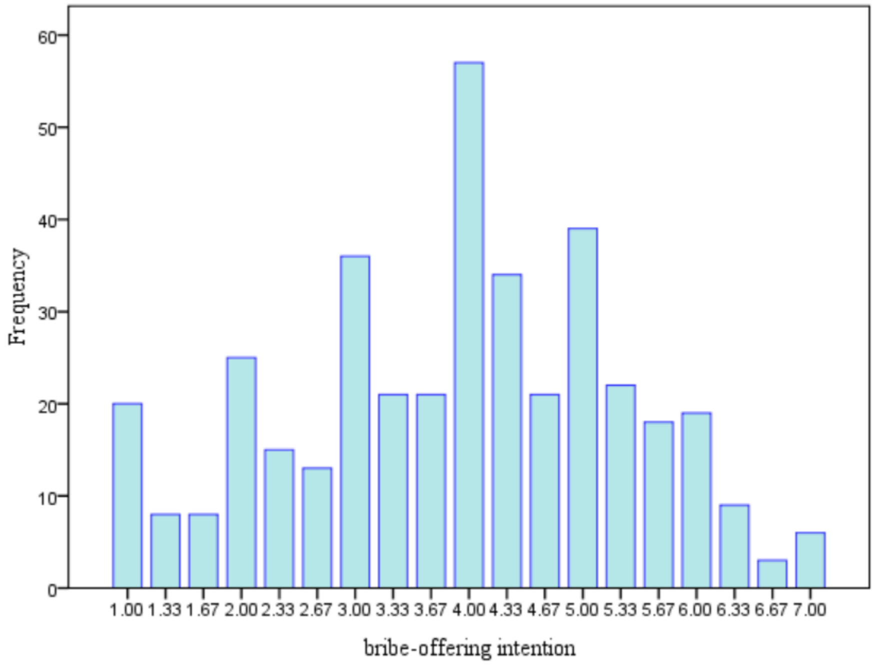

★ **Study 2:**

The data distribution of belief in good luck in avoiding penalty (M_{ean} = 3.56, M_{edian} = 3.33, M_{ode} = 1.00, SD = 1.72):

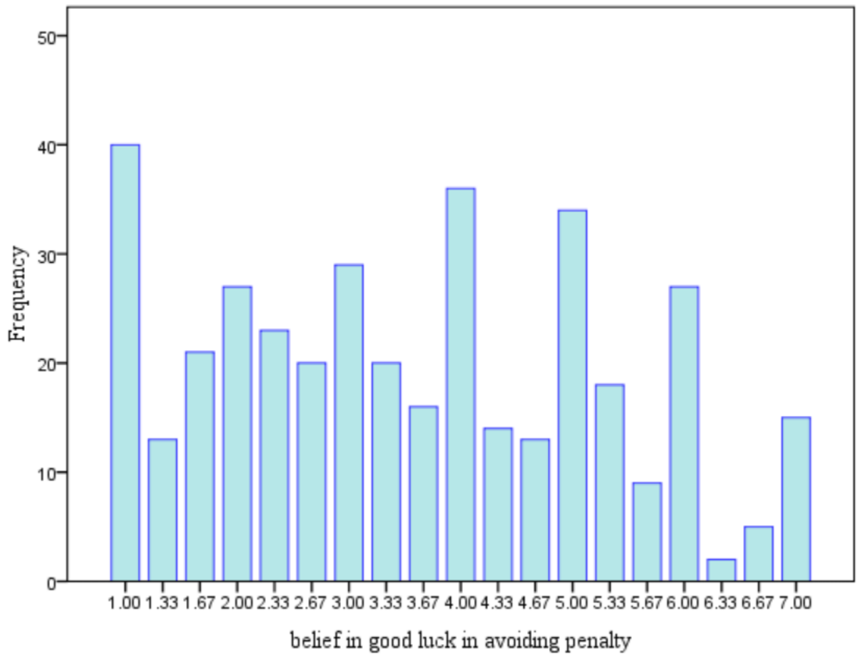

The data distribution of bribe-taking intention (M_{ean} = 3.09, M_{edian} = 3.00, M_{ode} = 1.00, SD = 1.56):

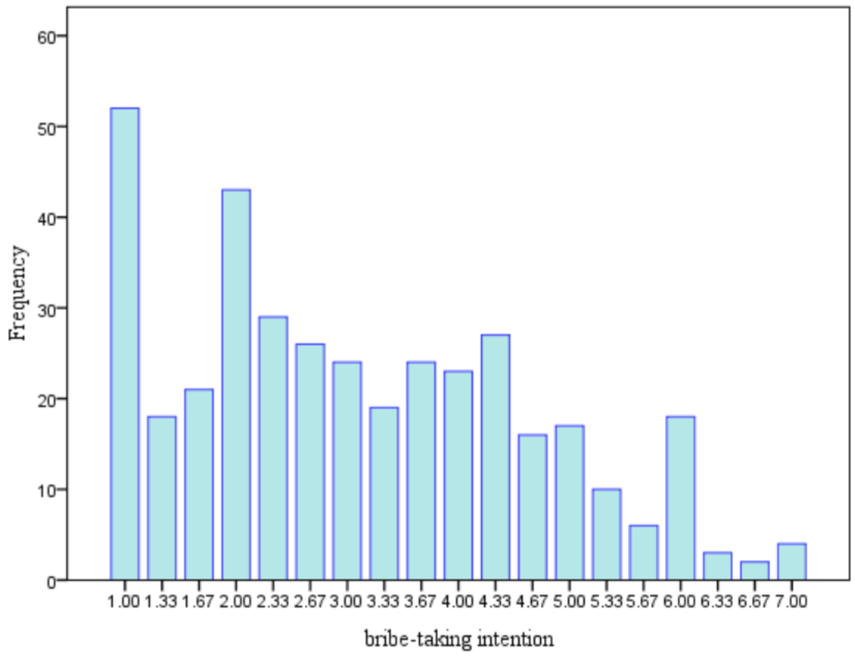

Supplement: Supplementary file 1 [file Presentation2.PDF]
